# Vitamin D Reshapes Genomic Hierarchies in Skin Cells: lncRNA-Driven Responses in Carcinoma Versus Transcription Factor-Based Regulation in Healthy Skin

**DOI:** 10.3390/ijms26146632

**Published:** 2025-07-10

**Authors:** Anna M. Olszewska, Joanna I. Nowak, Paweł Domżalski, Kamil Myszczyński, Michał A. Żmijewski

**Affiliations:** 1 Department of Histology, Medical University of Gdansk, 1a Debinki, 80-211 Gdansk, Polandmzmijewski@gumed.edu.pl (M.A.Ż.); 2 Centre of Biostatistics and Bioinformatics Analysis, Medical University of Gdansk, 1a Debinki, 80-211 Gdansk, Poland

**Keywords:** long non-coding RNAs, vitamin D_3_, squamous cell carcinoma

## Abstract

The active form of vitamin D_3_, 1,25(OH)_2_D_3_, exerts hierarchical control over gene expression, initially targeting transcription factors (TFs) that drive downstream responses. Here, we profile the transcriptional landscape of primary keratinocytes (HPEKp) and squamous cell carcinoma (SCC) cells in response to 1,25(OH)_2_D_3_, revealing a distinct shift in regulatory targets. While TFs accounted for 9.23% of differentially expressed genes (DEGs) in keratinocytes, this proportion dropped to 4.9% with prolonged exposure. In contrast, SCC cells displayed a five-fold reduction in TFs deregulation and a concurrent enrichment of long non-coding RNAs (lncRNAs), which comprised 22.25% of DEGs after 24 h treatment, with 81% upregulated. Integrative transcriptomic and in silico analyses showed that lncRNA induction was predominantly VDR-dependent, partially RXRA-dependent, and PDIA3-independent. Notably, 90% of deregulated lncRNAs were atypical for head and neck SCC. Several of these lncRNAs exhibit potential antitumor properties and may modulate SCC cell responsiveness to interferon-gamma (IFN-γ). In conclusion, these findings suggest that in SCC cells, the regulation of lncRNA expression—rather than transcription factor modulation—may represent a mechanism of the cellular response to 1,25(OH)_2_D_3_.

## 1. Introduction

Squamous cell carcinoma (SCC) is the second most common skin cancer, derived from keratinocytes. The occurrence of SCC is related to ultraviolet irradiation, chemical exposure, and viral infection. SCC develops rapidly, and most patients are diagnosed at advanced stages, while regional lymph node metastasis may lead to serious complications or even be fatal [1]. Multiple studies have shown that abnormal expression of long non-coding RNAs (lncRNAs) has an important impact on SCC initiation and progression [2,3,4]. For example, overexpressed HOX transcript antisense RNA (*HOTAIR*) promotes SCC proliferation, migration, and epithelial-mesenchymal transitions by competitively combining with miR-326 to regulate the expression of PRA1 domain family member 2 protein (*PRAF2*) [5]. Metastasis-associated lung adenocarcinoma transcript 1 (*MALAT1*) inhibits SCC apoptosis and induces cell migration and invasion by regulating the Wnt/beta-catenin signaling pathway [6]. It has also been reported that P38-inhibited cutaneous squamous cell carcinoma-associated lncRNA (*PICSAR*), specifically expressed by SCCs, but not by keratinocytes, played a carcinogenic role by regulating the mitogen-activated protein kinase/extracellular signal-regulated kinase (MAPK/ERK) signaling pathway and promotes migration by downregulating integrin expression [7]. Among the downregulated lncRNAs in SCC, TINCR ubiquitin domain containing (*TINCR*), LINC00520, and Growth Arrest Specific 5 (*GAS5*) have been characterized. Induced increases in *TINCR* expression activate differentiation processes [8]; LINC00520 inhibits invasion and metastasis by inhibiting epidermal growth factor receptor (EGFR) and inactivating the phosphoinositide 3-kinase/protein kinase B (PI3K-AKT) signaling pathway [9]; *GAS5* inhibits proliferation and promotes apoptosis [10]. Such lncRNAs can be noninvasively extracted from body fluids, tissues, and cells, and can be used as biomarkers to improve diagnosis, prognosis, and chemoresistance. They can also be considered potential new targets in treating SCC.

LncRNAs are heterogeneous groups of transcripts lacking coding potential, involved in regulating gene expression or modulating non-genomic processes. LncRNAs participate in gene regulation by interacting with DNA elements, transcription factors, or other RNAs that serve as decoys, scaffolds, competitors, or modulators of chromatin structure. The non-genomic action is mediated by binding to proteins to regulate their functioning. LncRNAs may express their biological function through an interaction with several proteins, and their structure may be stabilized by a polyadenylated tail (polyA), which can also be recognized by the cellular machinery for nuclear export and translation [11]. Only a small group of lncRNAs are well characterized in SCC, normal keratinocytes [12,13], and after chemotherapy or irradiation of SCC [14,15,16]. Since it was postulated that the active form of vitamin D_3_ (1,25(OH)_2_D_3_) enhances the efficacy of cytotoxic chemotherapy for SCC [17], it seems interesting to study the effect of 1,25(OH)_2_D_3_ on the expression of lncRNAs in SCC. On the other hand, there is also growing evidence that lncRNAs are involved in gene expression regulation, but not much is known about their impact on cellular responses to 1,25(OH)_2_D_3_.

The active form of vitamin D_3_ through the classical cellular pathway modulates the expression of a large set of genes by interaction with the vitamin D nuclear receptor (VDR) and coreceptors such as retinoid X receptor alpha (RXRA) [18]. Importantly, beyond its well-established role in calcium homeostasis, 1,25(OH)_2_D_3_ has also been shown to exhibit anticancer properties by inhibiting cell proliferation, inducing apoptosis, and modulating cell differentiation and immune responses in various cancer cell types [19,20]. In addition, the role of protein A3 disulfide isomerase (PDIA3) in the non-classical fast responses to 1,25(OH)_2_D_3_ has recently been intensively investigated [21,22]. Although the genomic regulation by 1,25(OH)_2_D_3_ involves a large number of genes, not all of them contain ligand-bound VDR response elements (VDREs), either near the promoter regions or in distant regulatory elements [23,24]. Furthermore, hierarchical regulatory network analysis of 1,25(OH)_2_D revealed that transcription factors involved in the 1,25(OH)_2_D_3_ response act as intermediate regulators of gene expression [23].

In this study, we focused on analyzing lncRNAs deregulated in response to 1,25(OH)_2_D in both healthy and cancerous keratinocytes and compared these findings with transcription factors similarly affected under the same conditions. The aim was to determine whether the propagation of the 1,25(OH)_2_D response signal in these two cell types is primarily mediated by transcription factors (differentially expressed transcription factors: DETs) or lncRNAs (differentially expressed long non-coding RNAs: DELs). Recent studies have suggested that VDREs are located near non-coding RNA genes [25,26,27] and that 1,25(OH)_2_D_3_ can modulate the expression of multiple lncRNAs in various tissues and cancer types, including ovarian [28], colorectal [29], breast cancer [30], and lung cancer [31], as well as in the skin [27] and endothelial progenitor cells [32].

## 2. Results

### 2.1. VDR-RXRA Nuclear Colocalization Occurs in Both Cell Types but Follows Distinct Dynamics in Carcinoma Versus Normal Keratinocytes

To investigate differences in the genomic responses of A431 and HPEKp cells to the active form of vitamin D_3_, two time points were selected for transcriptomic analysis. These time points were chosen based on the dynamics of VDR translocation to the nucleus upon ligand binding, as determined by initial immunofluorescence observations. Subsequently, the colocalization of nuclear VDR with its coreceptor RXRA was also assessed. The first transcriptomic time point was selected to coincide with the initial observation of VDR translocation to the cell nucleus (characterized by a strong immunofluorescence signal in the nuclei compared to the cytoplasm) and also corresponded to the highest level of VDR colocalization with the RXRA coreceptor. The second time point was selected based on the observed decrease in the VDR-RXRA colocalization coefficient, with the Pearson correlation value dropping below 0.2, indicating that at this stage, colocalization is highly limited or nearly absent.

**Figure 1:**
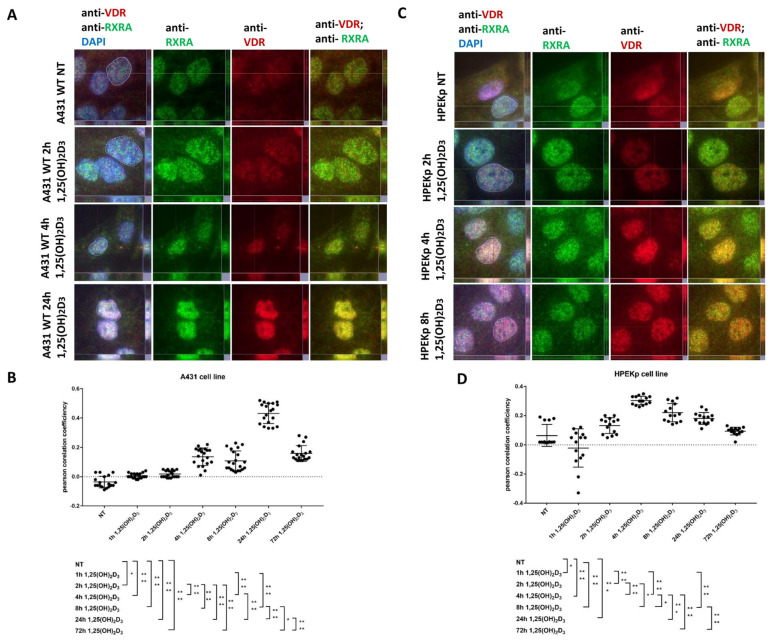
Analysis of VDR translocation and VDR-RXRA colocalization level after different 1,25(OH)_2_D_3_ time points treatment of squamous carcinoma cell line A431 WT, and primary culture keratinocytes HPEKp. (**A**) Images of A431 cells, non-treated and treated with 1,25(OH)_2_D_3_ at different time points, showing VDR fluorescence translocation and colocalization of VDR (anti-VDR; red) with RXRA protein (anti-RXRA; green) in nuclei (DAPI; blue). (**B**) Pearson’s correlation coefficient values for colocalization of VDR and RXRA in the nucleus of the A431 cells at different time points of 1,25(OH)_2_D_3_ treatment. (**C**) Images of HPEKp cells, non-treated and treated with 1,25(OH)2D3 at different time points, showing VDR fluorescence translocation and colocalization of VDR (anti-VDR; red) with RXRA protein (anti-RXRA; green) in nuclei (DAPI; blue). (**D**) Pearson’s correlation coefficient values for colocalization of VDR and RXRA in the nucleus of the HPEKp cells at different time points of 1,25(OH)2D3 treatment. To analyze colocalization in cell nuclei, they were marked with a white dashed line using a dedicated neural network (cellSense, Olympus, Tokyo, Japan). Statistically significant differences are denoted with asterisks: * *p* < 0.05, *** *p* < 0.005, **** *p* < 0.0001. Scale bar 5 μm.

To establish these time points for 1,25(OH)_2_D_3_ treatment, both cell lines were labeled with antibodies targeting the VDR receptor and the RXRA coreceptor. Previous studies published by our group demonstrated that treatment of A431 cells with 1,25(OH)_2_D_3_ led to VDR-RXRA colocalization at relatively late time points (24–72 h) [33]. In the present study, we revisited the dynamics of VDR translocation in A431 SCC cells following 1,25(OH)_2_D_3_ stimulation, analyzing both early and late incubation time points. Furthermore, primary HPEKp keratinocytes from healthy donors were used as an appropriate control. As shown in Figure 1A,B, in A431 cells, the strongest colocalization of VDR and RXRA fluorescence signals was detected 24 h post-treatment. Additionally, after 72 h of incubation with 1,25(OH)_2_D_3_, a decrease in the colocalization value between VDR and RXRA proteins was observed (Figure 1B). In contrast, in normal HPEKp keratinocytes, the strongest VDR immunofluorescence signal in the nuclei was observed at the 4-h time point (Figure 1C). Additionally, peak colocalization between VDR and RXRA occurred at 4 h following 1,25(OH)_2_D_3_ treatment, followed by a progressive decline at later time points (Figure 1D). Variations in the dynamics of VDR nuclear translocation and its colocalization with RXRA, observed between tumor cells and healthy keratinocytes, guided the selection of optimal time points for transcriptomic investigations.

### 2.2. 1,25(OH)_2_D_3_ Treatment of Squamous Cell Carcinoma Cells (A431) Leads to a Time-Dependent Increase in DELs and a Decrease in DETFs

To investigate potential differences in the number of DELs and DETs between A431 cells and HPEKp cells treated with 1,25(OH)_2_D_3_, we reanalyzed the transcriptome data from A431 cells previously published by our group [33] and performed new transcriptomic experiments for HPEKp cells. Using an absolute log2FC ≥ 0 and FDR < 0.05, DELs accounted for 22% of all DEGs at the 24 h time point. The proportion of upregulated and downregulated protein-coding DEGs was relatively balanced, with 57% upregulated and 43% downregulated at the 24 h time point [33]. In contrast, the distribution of DELs was markedly different: 81% of DELs were upregulated, while only 19% were downregulated. This pronounced disparity highlights the potential significance of lncRNAs in mediating the cancer cell response to 1,25(OH)_2_D_3_.

**Figure 2:**
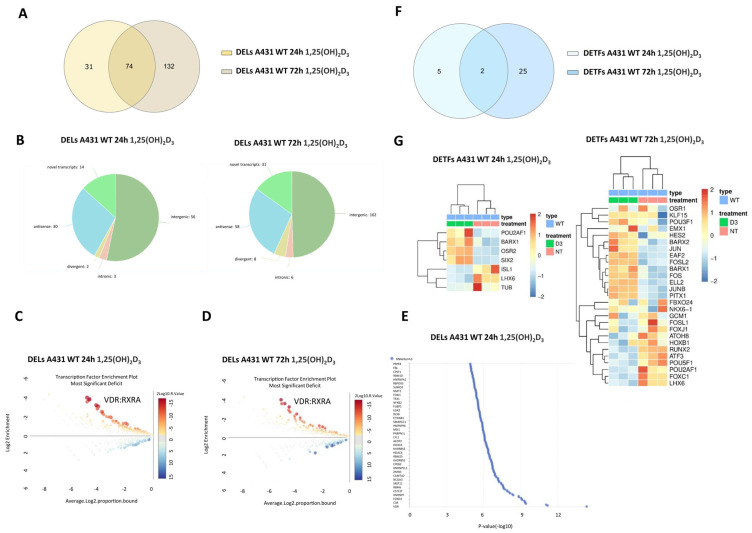
Time-dependent changes of differentially expressed lncRNAs (DELs) and differentially expressed transcription factors (DETFs) in a human squamous carcinoma cell line (A431) treated with 1,25(OH)_2_D_3_ for 24 h and 72 h. (**A**) Venn diagram showing the distribution of the DELs from A431 cells treated for 24 h or 72 h with 1,25(OH)_2_D_3_. (**B**) Classification of DELs from A431 cell line after 1,25(OH)_2_D_3_ treatment for 24 h and 72 h. (**C**,**D**) CiiiDER enrichment analysis showing the transcription factor binding sites (TFBSs) that are significantly over-represented (greater than zero) and under-represented (less than zero) for the DELs in A431 cell line after 1,25(OH)_2_D_3_ treatment for 24 h or 72 h compared with the background sequences. The plot shows the proportion of regions bound for each TF. The y-axis shows the enrichment (ratio of proportion bound), and the x-axis shows the average log proportion bound. The sizes and colors of the circles indicate the ±log10 (*p*-value). (**E**) LncSEA platform annotation analysis results show DEL-protein interactions in the A431 cell line after 1,25(OH)_2_D_3_ treatment for 24 h or 72 h. (**F**) Venn diagram showing the distribution of the DETFs in A431 WT cells treated for 24 h with 1,25(OH)2D3. (**G**) Heat map of RNA-seq expression data showing the DETFs in A431 WT cell line after 1,25(OH)_2_D_3_ treatment for 72 h.

The distribution of all DELs detected in A431 WT cells treated with 1,25(OH)_2_D_3_ for 24 h was shown as a Volcano plot (Figure S1A). Although the number of DELs affected by prolonged (72 h) treatment with 1,25(OH)_2_D_3_ of A431 WT cells nearly doubled (Venn diagram Figure 2A), the proportion of DELs relative to all DEGs only slightly decreased (from 22% after 24 h to 20.2% after 72 h). Interestingly, while the percentage of DELs relative to DEGs remained high, many upregulated DELs significantly decreased to 46% at the 72 h time point. All DELs detected in A431 WT cells treated with 1,25(OH)_2_D_3_ for 72 h were shown as a Volcano plot (Figure S1B). As much as 70% of the DELs from the 24 h time point were also deregulated after the prolonged incubation (72 h) with 1,25(OH)_2_D_3_. 77% of them showed only a limited time-dependent variability with absolute Δlog2FC < 0.5 (24 h vs. 72 h), and 23% showed significant time-dependent change with absolute Δlog2FC > 1 (24 h vs. 72 h).

In the next stage, the distribution of DELs on chromosomes was analyzed for both time points. (Figure S1C,D). The most mapped DELs at the 24 h time point were located on chromosomes 1, 3, and 12, indicating a heterogeneous distribution of DELs. A similar heterogeneous distribution of DELs was observed after extended incubation with 1,25(OH)_2_D_3_ (72 h); however, the majority of DELs were mapped to chromosomes 1, 3, and 6. The largest differences in abundance of DELs between the two time points were mapped to chromosomes 2, 11, and 14. Interestingly, all the chromosomes mentioned except chromosome 3 are not rearranged in squamous cell carcinoma [34]. Figure 2B demonstrates the percentage of the listed lncRNAs grouped based on their genomic position, orientation, and relative location to nearby protein-coding genes at both time points. It should be emphasized that this distribution was similar in both analyzed time points, and the majority of DELs were of the intergenic and antisense type.

To identify potential transcription factors responsible for regulating the expression of differentially expressed lncRNAs (DELs) at both time points, a transcription factor binding motif enrichment analysis was performed. The analysis revealed that the most enriched motif at the 24-h time point was MA0074.1, corresponding to the RXRA/VDR heterodimer (Enrichment: 5.21; Significance: 15.13) (Figure 2C). A similar result was observed at 72 h, with the RXRA/VDR motif again being the most enriched (Enrichment: 4.10; Significance: 10.41) (Figure 2D). RNA-protein interaction enrichment analyses performed in the lncSEA2.0 program demonstrated the putative interaction of DELs mainly with the VDR transcription factor (Figure 2E).

Analogous analyses were conducted for DETFs using the same thresholds (log2FC > 1 or log2FC < −1, and FDR < 0.05) (Figure 2F). Interestingly, DETFs represented only 1.5% of all DEGs at the 24 h time point. Prolonged incubation with 1,25(OH)_2_D_3_ (up to 72 h) resulted in a nearly three-fold increase in DETFs and a shift in the proportion between numbers of downregulated and upregulated DETFs (43% of upregulated at 24 h vs. 59% after 72 h) (Figure 2F). Interestingly, only two transcription factors were deregulated by 1,25(OH)_2_D_3_ at both time points. Figure 2G shows heat maps of all DETFs detected in A431 cells treated with 1,25(OH)_2_D_3_ for 24 h or 72 h. The gene coding Odd-skipped Related transcription factor 2 (*OSR2*) (log2FC = 1.12) was the most upregulated DETF at 24 h, while POU class 2 homeobox associating factor 1 (*POU2AF1*) was the most downregulated (log2FC = −1.87) at 24 h. The most upregulated DETF at 72 h was POU class 3 homeobox 1 (*POU3F1*) (log2FC = 4.47), and the most downregulated was *POU2AF1* (log2FC = −3.11). The DETF whose expression increased after prolonged incubation with 1,25(OH)_2_D_3_ (absolute Δlog2FC > 1, BM > 100) was BARX homeobox 1 (*BARX1*), and whose expression decreased was *POU2AF1*. The distribution of all DETFs detected in A431 cells treated with 1,25(OH)_2_D_3_ for 24 h and 72 h is shown as Volcano plots (Figure S1E,F). Due to the small number of transcription factors deregulated after 1,25(OH)_2_D_3_ treatment, to assess the number of RXRA/VDR motifs in promoter and distal regions, instead of an enrichment analysis, a classical mapping of the mentioned motifs was performed using the CiiiDER program. From both time points of 1,25(OH)_2_D_3_ treatment, only two transcription factors, Odd-Skipped Related Transcription Factor 2 (OSR2) and Homeobox Protein BarH-Like 1 (BARX1), had the mentioned motif detected within the ±500 kb region relative to the transcription start site (Table S1).

### 2.3. 1,25(OH)_2_D_3_ Treatment of Normal Keratinocytes (HPEKp) Leads to a Time-Dependent Decrease in DELs and an Increase in DETFs

**Figure 3:**
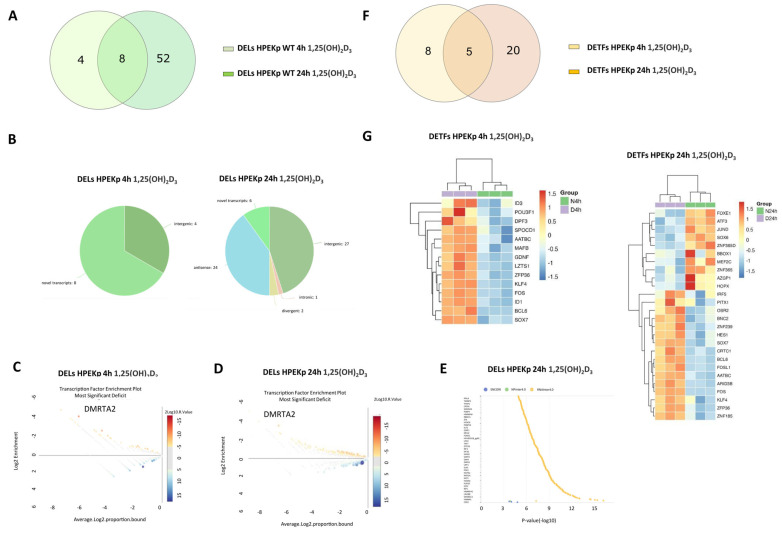
Time-dependent changes of differentially expressed lncRNAs (DELs) and differentially expressed transcription factors (DETFs) in normal keratinocytes (HPEKp) treated with 1,25(OH)_2_D_3_ for 4 h and 24 h. (**A**) Venn diagram showing the distribution of the DELs from HPEKp cells treated for 4 h or 24 h with 1,25(OH)_2_D_3_. (**B**) Classification of DELs from HPEKp cell line after 1,25(OH)_2_D_3_ treatment for 4 h and 24 h. (**C**,**D**) CiiiDER enrichment analysis showing the transcription factor binding sites (TFBSs) that are significantly over-represented (greater than zero) and under-represented (less than zero) for the DELs in HPEKp cell line after 1,25(OH)_2_D_3_ treatment for 4 h or 24 h compared with the background sequences. The plot shows the proportion of regions bound for each TF. The y-axis shows the enrichment (ratio of proportion bound), and the x-axis shows the average log proportion bound. The sizes and colours of the circles indicate the ±log10 (*p*-value). (**E**) LncSEA platform annotation analysis results show DEL-protein interactions in the HPEKp cell line after 1,25(OH)_2_D_3_ treatment for 24 h. (**F**) Venn diagram showing the distribution of the DETFs in HPEKp cells treated for 4 h and 24 h with 1,25(OH)_2_D_3_. (**G**) Heat map of RNA-seq expression data showing the DETFs in A431 WT cell line after 1,25(OH)_2_D_3_ treatment for 4 h and 24 h.

The transcriptomic analysis of HPEKp cells treated with 100 nM 1,25(OH)_2_D_3_ for 4 h revealed that DELs accounted for 8.5% of all DEGs compared to non-treated cells, and 99% of them were upregulated (Figure 3A). Prolonged incubation to 24 h resulted in slight changes in the proportion of DELs to all DEGs (an increase to 11.76%) (Figure 3A). Intestinally, the percentage of up- and downregulated DELs after 24 h also changed to 58% upregulated and 42% downregulated. As much as 66% of the DELs downregulated at 4 h were also deregulated after prolonged incubation (24 h) with 1,25(OH)_2_D_3_ (Figure 3A). 75% of them showed only a limited time-dependent variability with absolute Δlog2FC < 0.5 (4 h vs. 24 h), and 25% showed significant time-dependent change with absolute Δlog2FC > 1 (24 h vs. 72 h). In the next stage, the chromosomal distribution of DELs from two time points was analyzed (Figure S2A,B). The most mapped DELs at the 4 h time point were found on chromosomes 2, 11, and 19, indicating a heterogeneous distribution of DELs. A similar heterogeneous distribution of DELs was observed in the case of extended incubation with 1,25(OH)_2_D_3_ up to 24 h. However, most DELs were mapped to chromosomes 10, 17, and 19 after prolonged treatment. The largest time-dependent differences in the distribution of DELs were observed on chromosomes 2, 11, and 14. Figure 3B demonstrates the percentage of the listed lncRNAs grouped based on their genomic position, orientation, and relative location to nearby protein-coding genes at both time points. It should be emphasized that this distribution was different in both analyzed time points, and most DELs were of the novel transcripts and intergenic type.

To check the potential involvement of transcription factors in the regulation of DELs expression, transcription factor enrichment analysis was performed. It was shown that MA1478.1 for DM-type intertwined zinc finger factors (DMRTA2) was the most enriched motif (for 4 h: Enrichment 7.23; Significance 1.94; for 24 h: Enrichment 7.23; Significance 1.94) and POU Class 4 Homeobox 2 (POU4F2) the most enriched at 24 h time-point treatment (: Enrichment 7.32; Significance 1.74 (Figure 3C,D). Next, RNA-protein interaction enrichment analyses performed in the lncSEA2.0 program demonstrated the putative interaction of DELs mainly with the Caudal Type Homeobox 2(CDX2) transcription factor and heterogeneous nuclear ribonucleoprotein L (HNRNPL) (Figure 3E). This result may indicate VDR-independent transcriptional regulation of DELs expression in healthy skin cells after 1,25(OH)_2_D_3_ administration.

Analogous analyses were conducted for DETFs at the same time points of 1,25(OH)_2_D_3_ treatment and the same thresholds (log2FC > 1 or log2FC < −1, and FDR < 0.05). DETFs accounted for 9.23% of all DEGs compared to non-treated cells (log2FC > 1 or log2FC < −1, and FDR < 0.05). These DETFs were all upregulated in the upward direction. Extending the incubation time with 1,25(OH)_2_D_3_ to 24 h resulted in an over 3.5-fold increase in the total number of DEGs and, interestingly, a two-fold decrease in the proportion of DETFs (4.9%) (Figure 3F). Such prolonged incubation with 1,25(OH)_2_D_3_ also changes the proportion between DETFs, up and down deregulated, from upregulation at 4 h to balanced up and downregulation at the 24 h time point.

Interestingly, only 20% of identified DETFs at the 24 h time point were common for both time points (Figure 3F). The four of them (*BCL6*, *KLF4*, *SOX7*, *ZFP36*) showed a limited time-dependent variability with absolute Δlog2FC < 0.35 (4 h vs. 24 h), and only FOS expression increased due to longer incubation with 1,25(OH)_2_D_3_ (FOS: Δlog2FC = 1.22). Figure 3G shows a heat map with all DETFs detected in HPEKp keratinocytes treated with 1,25(OH)_2_D_3_ for 4 h or 24 h.

Similarly, due to the small number of transcription factors identified after 1,25(OH)_2_D_3_ treatment, a classical mapping of RXRA/VDR motifs in promoter and distal regions was again performed using the CiiiDER program. As in the previous experiment, only two transcription factors, OSR2 and IRF5 (Interferon regulatory factor 5), had the RXRA/VDR motif detected within the ±500 kb region relative to the transcription start site (Table S2).

### 2.4. VDR-Dependent DEL and DET Responses to 1,25(OH)_2_D_3_ in A431 Cells Are Abolished by VDR Knockout and Diverge from Typical HNSCC Patterns

**Figure 4:**
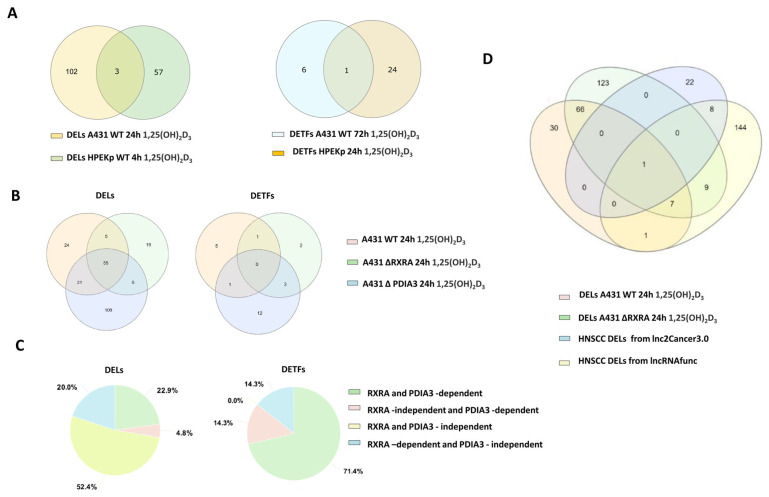
Comparative analysis of DELs from 1,25(OH)2D3-treated A431 cells to DELs from healthy cells, A431 cells lacking functional VDR, RXRA, and PDIA3 genes, and lncRNAs typical of head and neck squamous cell carcinoma (HNSC). (**A**) Venn diagrams showing the differences in DELs and DETs between healthy cells and cancerous cells treated with 1,25(OH)2D3. (**B**) Venn diagrams showing the distribution of the DELs and DETFs in A431 WT, A431 ΔRXRA, and A431 ΔPDIA3 cells treated for 24 h with 1,25(OH)2D3. (**C**) Percentage distribution of RXRA dependent/independent genes and/or PDIA3 after 24 h 1,25(OH)2D3 treatment. (**D**) Venn diagram showing the distribution of the DELs between DELs from HNSC databases lnc2Cancer3.0 and lncRNAfunc and DELs from A431 cells treated with 1,25(OH)2D3 for 24 h and 72 h.

A direct comparison of DELs and DETs between healthy keratinocytes and squamous carcinoma cells following treatment with 1,25(OH)_2_D_3_ revealed substantial differences, as illustrated in Figure 4A. Subsequently, the study explored the potential involvement of VDR, its heterodimeric partner retinoid X receptor alpha (RXRA), and the non-genomic receptor protein disulfide isomerase family A member 3 (PDIA3)—a mediator of rapid cellular responses to 1,25(OH)_2_D_3_—in regulating the expression of lncRNAs and transcription factors in A431 cells. For this purpose, A431 cells with CRISPR-mediated knockouts of functional *VDR*, *RXRA,* or *PDIA3* genes were used [21,33]. In our previous study, after 24 h incubation with 1,25(OH)_2_D_3_ cells lacking VDR protein (A431Δ*VDR*), no DEGs were detected compared to untreated cells (threshold log2FC > 1 and FDR < 0.05) [33]. Although extended incubation (72 h) showed the presence of 20 DEGs (log2FC > 1 and FDR < 0.05), none of the DEGs coded for lncRNAs or TFs. In the case of A431Δ*RXRA* cells at the 24-h time point of treatment, 21.7% of all detected DEGs coded DELs (70% upregulated and 30% downregulated)**.** The distribution of all DELs was shown as a Volcano plot (Figure S3A). It should be emphasized that the percentage distribution of DELs in A431Δ*RXRA* cells treated with 1,25(OH)_2_D_3_ for 24 h was quite similar to results from A431WT cells treated for 24 h or 72 h. In the case of A431Δ*PDIA3* cells, among all DEGs, 27% of DELs were identified (61% upregulated and 39% downregulated). A distribution of all DELs was shown as a Volcano plot (Figure S3B). The percentage distribution of up- and downregulated DELs was also similar to the percentage distribution of DELs from the 24 h time point, 72 h in A431 WT cells and A431Δ*RXRA* cells treated with 1,25(OH)_2_D_3_ for 24 h.

As expected, no transcription factors (DETFs) were found in A431Δ*VDR* cells treated with 1,25(OH)_2_D_3_ (both time points). In A431Δ*RXRA* cells, only six DETFs were found, including four upregulated (*OSR2, MAF, KLF15, EAF2*), and two downregulated were identified (*RUNX2, RAET1L*)**.** In the case of A431Δ*PDIA3* cells, 16 DETFs, including 9 upregulated and 7 downregulated, were identified. To elucidate the impact of VDR, RXRA, and PDIA3 proteins on the genomic response to 1,25(OH)_2_D_3,_ the expression profiles of DELs and DETFs in A431 cells were compared with those of the A431Δ*VDR*, A431Δ*RXRA, and* A431Δ*PDIA3* cells for both time points. Figure 4B shows a Venn diagram with the clustering of the DELs and DETs amongst all studied groups, except A431Δ*VDR* cells treated with 1,25(OH)_2_D_3_ for 24 h. As no DEG was detected in A431Δ*VDR* cells treated with 1,25(OH)_2_D_3_, it could be postulated that DELs and DETFs expression is also VDR-dependent in A431 cells. Interestingly, under identical conditions, only 42,8% of DELs were found to be RXRA-dependent, while the expression of the majority (85.74%) of DETFs was dependent on RXRA protein. In the case of PDIA3 protein knockout, only 27.6% DELs were found to be PDIA3-dependent, while the presence of PDIA3 was essential for the expression of 85.74% DETFs, and 51.5% DEGs were PDIA3-dependent (Figure 4C). All annotations found in PubMed regarding DELs from A431 cells treated with 1,25(OH)_2_D_3_ were summarized in Table 1 and Table 2.

It is well established that 1,25(OH)_2_D_3_ possesses anticancer properties [35,36,37,38,39,40,41] while dysregulated lncRNA expression may lead to the promotion or suppression of cancer development [42]. Thus, in the next stage of the project, we investigated whether the lncRNAs deregulated after 1,25(OH)_2_D_3_ treatment in the A431 squamous cell carcinoma cell line overlapped with lncRNAs previously identified in head and neck squamous cell carcinoma (HNSCC). To this end, the list of DELs detected in 1,25(OH)_2_D_3_-treated A431 WT cells was compared with the set of lncRNAs known to be deregulated in HNSCC. The online tools lnc2Cancer 3.0 and lncRNAfunc were used to analyze tumor RNA-seq expression data. Subsequently, the DELs characteristic of HNSCC and those affected by 1,25(OH)_2_D_3_ treatment in A431 WT cells were compared and visualized using Venn diagrams (Figure 4D). Interestingly, 91% of DELs detected after treatment of A431 cells with 1,25(OH)_2_D_3_ for 24 h or 72 h were not deregulated in HNSCs. Of the remaining DELs from the 24 h or 72 h time points, 14 DELs (out of 18 in common) were upregulated, as in the case of HNSCCs, 1 DEL was downregulated, as in HNSCs, and the remaining 3 lncRNAs showed the opposite direction of change in expression compared to HNSCs. Notably, most of the DELs from the 24 h and 72 h time points that occur in HNSCs turn out to be independent of RXRA or PDIA3. In summary, 91% and 96% of the DELs identified in A431 cells after 24 and 72 h of 1,25(OH)_2_D_3_ treatment, respectively, were not previously reported as characteristic for head and neck squamous cell carcinoma, suggesting that the majority of the 1,25(OH)_2_D_3_–responsive lncRNAs in this model are not typically associated with HNSCC.

**Table 1:** RXRA-dependent differentially expressed lncRNAs and TFs.

Gene ID	Expression Level in A431 Treated with 1,25(OH)_2_D_3_/PDIA3-Dependency	Role in Different Cancers	Ref.
*LINC00973*	Upregulated only at 24 h treatment (PDIA3-independent)	Promotes the Warburg effect by enhancing LDHA enzyme activity in breast cancer	[43]
		Knockdown of LINC0973 decreases p21 levels, activates the cellular proliferation of cancer cells, and suppresses the apoptosis of drug-treated colorectal cancer	[44]
		Involved in cancer immune suppression through positive regulation of Siglec-15 (sialic acid binding Ig like lectin) in renal cell carcinoma	[45]
		The expression of LINC00973 is consistently increased upon treatment of colon cancer cells	[46]
*TONSL-AS1*	Upregulated only at 24 h treatment (PDIA3-dependent)	TONSL-AS1 regulates the progression of gastric cancer by activating TONSL	[47]
		Overexpression of TONSL-AS1 resulted in the upregulation of CDK1 and poor prognosis (ovarian cancer)	[48]
		MicroRNA-135a expression is upregulated in hepatocellular carcinoma and targets long non-coding RNA TONSL-AS1 to suppress cell proliferation	[49]
*LINC01764*	Upregulated at 24 h and 72 h treatment (PDIA3-independent)	Low expression of LINC01764 was associated with poor prognoses in bladder cancer patients	[50]
		LINC01764, based on RNA interactions, regulates UCA1 expression in the prevention of colorectal cancer	[51]
*LINC00880*	Upregulated only at 24 h treatment (PDIA3-dependent)	Higher expression of LINC02086 and LINC00880 predicted worse overall survival in hepatocellular carcinoma	[52]
*LINC01559*	Upregulated at 24 h and 72 h treatment (PDIA3-independent)	Promotes progression of gastric cancer via the PI3K/AKT signaling pathway	[53]
		LINC01559 promotes colorectal cancer via sponging miR-1343-3p to modulate PARP1/PTEN/AKT pathway	[54]
		LINC01559 accelerates pancreatic cancer cell proliferation and migration through the YAP-mediated pathway	[55]
		Promotes resistance of hepatocellular carcinoma to oxaliplatin by directly sponging miR-6783-3p	[56]
		Indicates lymph node metastasis and poor prognosis Promotes lung cancer cell proliferation and migration in vitro, by enhancing the autophagy signal pathway via sponging has-miR-1343-3p	[57]
		Knockdown of LINC01559 inhibited breast cancer cell proliferation, migration, and invasion	[52]
		Promotes pancreatic cancer progression by acting as a competing endogenous RNA of miR-1343-3p to upregulate RAF1 expression	[58]
*CALML3-AS1*	Upregulated at 24 h and 72 h treatment (PDIA3-dependent)	Suppresses papillary thyroid cancer progression via sponging miR-20a-5p/RBM38 axis Promotes the tumorigenesis of bladder cancer via regulating ZBTB2 by suppression of microRNA-4316 Upregulation of CALML3-AS1 promotes cell proliferation and metastasis in cervical cancer via activation of the Wnt/β-catenin pathway	[59]
			[60]
			[61]
*LINC01193*	Downregulated at 24 h and 72 h treatment (PDIA3-independent)	Overexpression predicts the tumor stage and patient survival rate in esophageal squamous cell carcinoma	[62]
*ITGB2-AS1*	Downregulated at 24 h and 72 h treatment (PDIA3-independent)	Promotes the progression of clear cell renal cell carcinoma by modulating the miR-328-5p/HMGA1 axis	[63]
		Promotes the migration and invasion of breast cancer cells through upregulating ITGB2	[64]
		Promotes cisplatin resistance of non-small cell lung cancer by inhibiting ferroptosis via activating the FOSL2/NAMPT axis	[65]
		Downregulation of ITGB2-AS1 inhibits osteosarcoma proliferation and metastasis by repressing Wnt/β-catenin signalling	[66]

**Table 2:** RXRA-independent differentially expressed lncRNAs and TFs.

Gene ID	Expression Level in A431 Treated with 1,25(OH)_2_D_3_/PDIA3-Dependency	Role in Different Cancers	Ref.
*NPSR1-AS1*	upregulated at 24 h and 72 h treatment (PDIA3-independent)	promotes the proliferation and glycolysis of hepatocellular carcinoma cells by regulating the MAPK/ERK pathway	[67]
		activates the MAPK pathway to facilitate thyroid cancer cell malignant behaviors via recruiting ELAVL1 to stabilize NPSR1 mRNA	[68]
		expressions of NPSR1-AS1 were negatively associated with CD8 T cells in lung adenocarcinoma	[69]
*UCA1*	upregulated at 24 h and 72 h treatment (PDIA3-independent)	UCA1 from cancer-associated fibroblasts enhances chemoresistance in vulvar squamous cell carcinoma cells	[70]
		promotes proliferation and cisplatin resistance of oral squamous cell carcinoma by suppressing miR-184 expression	[71]
		promotes cell proliferation, invasion, and migration of laryngeal squamous cell carcinoma cells by activating the Wnt/β-catenin signaling pathway	[72]
		regulates CCR7 expression to promote tongue squamous cell carcinoma progression by sponging miR-138-5p	[73]
		inhibits esophageal squamous-cell carcinoma growth by regulating the Wnt signaling pathway	[74]
*AATBC*	upregulated at 24 h and 72 h treatment (PDIA3-independent)	promotes the proliferation and migration of prostate cancer cells through the miR-1245b-5p/CASK Axis	[75]
		facilitates the cell growth and metastasis of cervical cancer as a sponge of miR-1245b-5p	[76]
		promotes breast cancer migration and invasion by interacting with YBX1 and activating the YAP1/Hippo signaling pathway	[77]
		suppresses proliferation and induces apoptosis in bladder cancer	[78]
*LINC01748*	upregulated at 24 h and 72 h treatment (PDIA3-independent)	cancerogenic role in lung cancer via the microRNA-520a-5p/HMGA1 axis regulation	[79]
*LINC02474*	upregulated at 24 h and 72 h treatment (PDIA3-independent)	upregulation of LINC02474 promotes migration and invasion of colorectal cancer cells	[80]
*TRIM31-AS1*	upregulated only at 24 h treatment (PDIA3-independent)	highly expressed in colon organoids	[81]
*LINC00649*	upregulated at 24 h and 72 h treatment (PDIA3-independent)	promotes the development of bladder cancer by regulating the miR-15a-5p/HMGA1 axis	[82]
		promotes the development of breast cancer via the stabilization of HIF-1α through the NF90/NF45 complex	[83]
		promotes the development of lung squamous cell carcinoma via activating the MAPK signaling pathway	[84]
*FLG-AS1*	upregulated at 24 h and 72 h treatment (PDIA3-independent)	downregulated in oral SCC	[85]
*LINC02428*	upregulated at 24 h and 72 h treatment (PDIA3-independent)	overexpressed LINC02428 suppressed the proliferation and metastasis of HCC	[86]
*CASC9*	upregulated at 24 h and 72 h treatment (PDIA3-independent)	promotes esophageal squamous cell carcinoma metastasis through upregulating LAMC2 expression	[87]
		promotes tumor progression by suppressing autophagy-mediated cell apoptosis via the AKT/mTOR pathway	[88]
		promotes tumorigenesis by affecting epithelial-mesenchymal transition	[89]
*LINC00491*	upregulated at 24 h and 72 h treatment (PDIA3-independent)	promotes proliferation and inhibits apoptosis	[90]
*LINC01605*	upregulated at 24 h and 72 h treatment (PDIA3-independent)	promotes the proliferation of laryngeal squamous cell carcinoma	[91]
		promotes tumor growth in nasopharyngeal carcinoma via regulation of the NF-κB pathway	[92]
*BBOX1-AS1*	upregulated at 24 h and 72 h treatment (PDIA3-independent)	BBOX1-AS1 silencing inhibits esophageal squamous cell cancer progression	[93]
		promotes esophageal squamous cell carcinoma by regulating the HOXB7/β-catenin axis	[94]
		promotes esophageal squamous cell carcinoma by activation of the Hedgehog signaling pathway	[95]
*MIR4713HG*	upregulated at 24 h and 72 h treatment (PDIA3-independent)	aggravates malignant behaviors in oral tongue squamous cell carcinoma via binding with microRNA let-7c-5p	[96]
*LINC00243*	downregulated at 24 h and 72 h treatment (PDIA3-independent)	promotes proliferation and glycolysis in non-small cell lung cancer cells	[97]

### 2.5. Deregulated lncRNAs Following 1,25(OH)_2_D_3_ Treatment Can Be Potentially Involved in Modulating Interferon-Gamma Signaling Pathways

**Figure 5:**
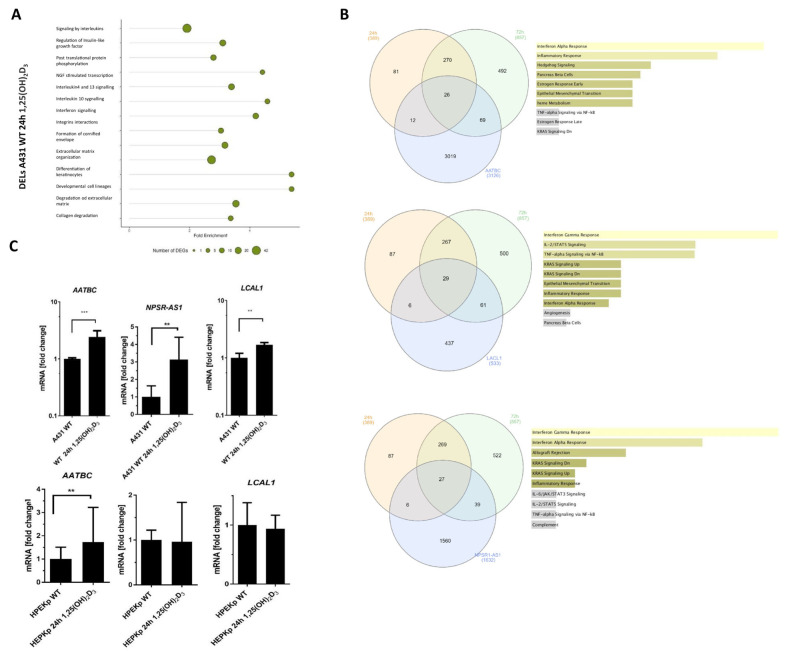
The possible impact of lncRNAs on interferon-gamma signaling after 1,25(OH)_2_D_3_ treatment of squamous cell carcinoma line A431. (**A**) Reactome analysis of DELs in A431 cells treated with 1,25(OH)_2_D_3_ for 24 h. (**B**) The Venn diagrams show AATBC, NPSR1-AS, and LCAL1 target genes (downloaded from the lncRNAfunc tool) with all DEGs from A431 cells treated with 1,25(OH)_2_D_3_ for 24 h and 72 h, and the ontology of genes is common to the three defined groups mentioned above. (**C**) mRNA expression of AATBC, NPSR1-AS1, LCAL1 before and after treated with 1,25(OH)2D3 of A431 and HPEKp cells. Statistically significant differences are denoted with asterisks: ** *p* < 0.01, *** *p* < 0.005.

To identify the biological pathways potentially involving the DELs detected after 1,25(OH)_2_D_3_ treatment of A431 cells, we performed a detailed ORA Reactome analysis. This analysis revealed significant enrichment in processes such as ‘Extracellular matrix organization’ (gene ratio 38/515), ‘Degradation of the extracellular matrix’ (gene ratio 23/515), and ‘Interferon signaling’ (gene ratio 15/515) (Figure 5A, Supplementary Table S3). Next, we selected specific DELs and retrieved their target genes from the lncRNAfunc database. We then compared these targets with the lists of genes deregulated in A431 cells after 1,25(OH)_2_D_3_ treatment for 24 and 72 h, respectively. Finally, we performed gene ontology analysis on the set of genes overlapping across all three groups: DEL targets, 24-h deregulated genes, and 72-h deregulated genes. Figure 5B illustrates the results of the gene ontology analysis for these shared genes. In all groups, the genes were predominantly associated with the cellular response to interferon signaling (Figure 5B). To validate the transcriptomic findings, real-time PCR analysis was conducted. Among the significantly upregulated lncRNAs following 24 h of 1,25(OH)_2_D_3_ treatment were AATBC, NPSR1-AS1, and LCAL1 (Figure 5C). Analogous experiments in control keratinocytes showed that only AATBC expression increases after 1,25(OH)_2_D_3_ treatment, while the expression of NPSR-AS1 and LCAL1 remains unchanged (Figure 5C).

### 2.6. Gene Ontology and Gene Set Enrichment Analysis of All 1,25(OH)2D3–Deregulated Genes in A431 Cells Revealed a Significant Enrichment in Pathways Related to the Interferon-Gamma Response

Previously, we observed that lncRNAs responsive to 1,25(OH)_2_D_3_ might regulate genes involved in interferon-related pathways. However, due to limited data on lncRNAs in the literature and the scarcity of online tools for reliable functional prediction, we extended our analysis to include all genes deregulated by 1,25(OH)_2_D_3_ treatment in A431 cells. The aim was to assess whether their deregulation could significantly impact interferon-associated processes, with particular attention to DEGs observed after 72 h of 1,25(OH)_2_D_3_ incubation, as these may be regulated by DELs identified at the 24-h time point.

**Figure 6:**
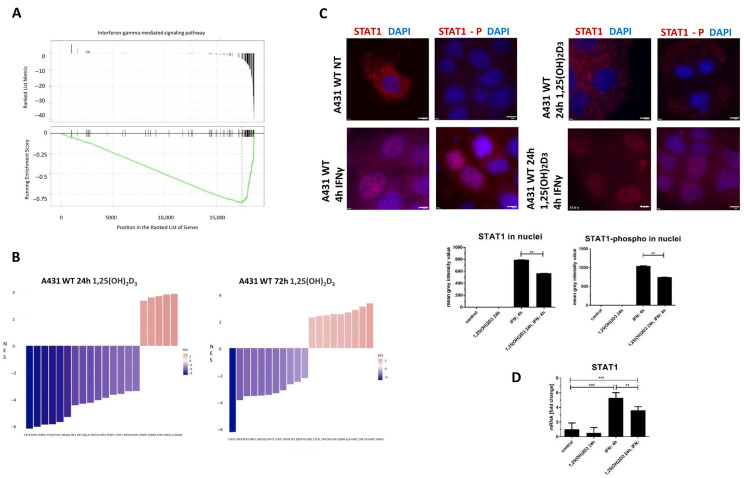
The impact of 1,25(OH)_2_D_3_ on interferon-gamma-mediated signaling pathway. (**A**) GSEA analyses of the DEGs after 72 h 1,25(OH)_2_D_3_. (**B**) Top 20 transcription factors showing the greatest changes compared to A431 1,25(OH)_2_D_3_ treated for 24 h or 72 h vs. non-treated cells. Negative values indicate a decrease in expression of 1,25(OH)_2_D_3_ compared to non-treated A431 cells, while positive values indicate an increase (analyses made on all DEGs). (**C**) Images of A431 cells (control, non-treated with 1,25(OH)_2_D_3_; NT) treated with 1,25(OH)_2_D_3_ for 24 h, with IFNγ for 4 h, or pretreated with 1,25(OH)_2_D_3_ for 24 h. Images show: STAT1 (red) or STAT1-phospho (red) with DAPI (blue) signals. The lower panel shows mean grey intensity values (fluorescence intensity) of STAT1 and STAT1-phospho in A431 cells at different treatment combinations. (**D**) mRNA expression pattern of STAT1 treated with 1,25(OH)_2_D_3_ and IFNγ at different combinations. Statistically significant differences are denoted with asterisks: ** *p* < 0.01, *** *p* < 0.005. Scale bar 5 μm.

Gene ontology analyses for both 24- and 72-h incubation periods revealed enrichment of terms such as ‘positive regulation of inflammatory response’ (GO:0050729) and ‘interferon-gamma-mediated signaling pathway’ (GO:0060333), consistent with the well-established immunomodulatory effects of 1,25(OH)_2_D_3_ (Figure S4). Additionally, GSEA of DEGs from the 72-h treatment showed that genes associated with the “interferon-gamma-mediated signaling pathway” were predominantly enriched at the bottom of the ranked gene list, indicating coordinated downregulation of this pathway. The negative enrichment score further supports the notion that 1,25(OH)_2_D_3_ may actively suppress interferon-gamma signaling (Figure 6A). Heatmaps illustrating genes involved in this pathway are presented in Figure S5.

In line with these findings, GSEA confirmed that the majority of genes in the interferon-gamma (IFNγ) signaling pathway were downregulated after both 24 and 72 h of 1,25(OH)_2_D_3_ treatment. Given that STAT1 (Signal transducer and activator of transcription 1) is a key transcription factor and central mediator of the IFNγ signaling cascade, it was selected for further investigation. Interestingly, additional analysis of transcription factor expression—performed without applying an FDR threshold—revealed a reduction in STAT1 expression at both time points (Figure 6B). To confirm the potential involvement of 1,25(OH)_2_D_3_ in the downregulation of the response to IFNγ, immunofluorescent detection of STAT1 and qPCR were performed. Figure 6C shows the immunofluorescent staining of STAT1 and phosphorylated STAT1-phospho (on Tyr 701) in A431 cells, non-treated and treated with IFNγ in the presence or absence of 1,25(OH)_2_D_3_. As expected, treatment with IFNγ for 4 h increased the fluorescence intensity of both STAT1 and STAT1-phospho, and a signal was visible in the nuclei. After treatment with 1,25(OH)_2_D_3_, the fluorescence intensity of STAT1 decreased in A431 cells, while STAT1-phospho was undetectable. Interestingly, pretreatment with 1,25(OH)_2_D_3_ (for 24 h) and subsequent treatment of cells with IFNγ for 4 h reduces translocation of STAT1 and STAT1-phospho into the nucleus, when compared to cells treated only with IFNγ (Figure 6C). Additionally, qPCR showed that the expression of the *STAT1* gene is reduced by 1,25(OH)_2_D_3_ pretreatment in A431 cells subjected to IFNγ (Figure 6D).

## 3. Discussion

The first study suggesting a role for VDR-dependent lncRNAs in skin cancer protection was published in 2014 by Bikle and colleagues [27,98]. In that work, the relationship between lncRNAs and VDR was explored by analyzing lncRNA expression in VDR-deficient mouse keratinocytes. They found increased levels of pro-cancer lncRNAs in these cells, suggesting that loss of VDR creates an imbalance that may predispose keratinocytes to tumorigenesis [27]. In our study, we investigated the effects of 1,25(OH)_2_D_3_ on lncRNA expression in the human squamous cell carcinoma line A431 and compared the results to primary human keratinocytes (HPEKp). Consistent with our findings, previous research has demonstrated that VDR is expressed in head and neck squamous cell carcinoma (HNSCC) tumors, and that adding 1,25(OH)_2_D_3_ to chemotherapy can reduce tumor growth and cell migration [99].

We observed a pronounced disproportion in the regulation of transcription factors and lncRNAs following 1,25(OH)_2_D_3_ treatment, with clear differences between healthy and cancerous cells. Notably, A431 cells exhibited a substantially greater number of deregulated lncRNAs, suggesting a distinct role for these molecules in mediating the cellular response to 1,25(OH)_2_D_3_ signaling. We hypothesize that this disproportion may reflect an active role of lncRNAs in amplifying and sustaining the genomic effects of 1,25(OH)_2_D_3_, akin to the “second-wave” transcriptional response described for transcription factors [23]. Given the well-established roles of lncRNAs in regulating gene expression—via mechanisms such as chromatin remodeling, transcriptional interference, and post-transcriptional regulation—it is plausible that lncRNAs contribute significantly to the longer-term and cell type-specific outcomes of 1,25(OH)_2_D_3_ signaling. Importantly, after 24 h of incubation with 1,25(OH)_2_D_3_, 81% of differentially expressed lncRNAs were upregulated, underscoring the potential role of these molecules in modulating gene expression and protein function at this time point.

To date, characterization of indirect 1,25(OH)_2_D_3_–regulated genes has been limited to THP-1 (human monocytic) cells, with analyses focusing solely on transcription factors and restricted to the 24-h time point. It was suggested that 1,25(OH)_2_D_3_, via the VDR/RXRA receptor complex, stimulates the expression of TFs, which in turn, regulate the expression of further sets of genes that constitute a second, indirect cellular response to 1,25(OH)_2_D_3_. Nurminen et al. described four TFs: B-cell CLL/lymphoma 6 (BCL6), nuclear factor erythroid 2 (NFE2), E74-like ETS transcription factor 4 (ELF4), and POU class 4 homeobox 2 (POU4F2) as the secondary genomic effectors of 1,25(OH)_2_D_3_ treatment in acute monocytic leukemia THP-1 cells [100]. Interestingly, more accurate experiments showed that 1,25(OH)_2_D_3_ treatment of the THP-1 cell line for 2.5 h, 4 h, and 24 h resulted in an upregulation of the 47 TFs [23]. However, our initial analysis of the transcriptome of A431 WT cells treated with 1,25(OH)_2_D_3_ showed deregulation of only a few TFs, of which only BARX1 was found to be involved in the secondary response to 1,25(OH)_2_D_3_ in THP-1 cells. The rest of the 1,25(OH)_2_D_3_-deregulated TFs (up- and downregulated) turned out to be specific to A431 cells only. It has to be acknowledged that our study was based on 24 h and 72 h time points; thus, it involved longer incubation times when compared to the THP-1 study [23]. In our bioinformatics analyses, we attempted to test whether the TFs deregulated after 1,25(OH)_2_D_3_ administration for 24 h could regulate the expression of genes at the 72 h time point. Unfortunately, the analysis (gene set enrichment on CiiiDER) showed that TFs deregulated in A431WT cells treated with 1,25(OH)_2_D_3_ for 24 h did not appear in the TFs regulating expression after prolonged incubation (72 h). Nevertheless, the analyses presented here suggest that transcription factors may have a reduced role in activating the second response to 1,25(OH)2D3 in skin cancer cells compared to THP-1 cells. In contrast, it seems that lncRNA may have a significant regulatory role in skin cancer cells, at least partially replacing TFs. The following facts support this conclusion: (1) less than 2% of all DEGs after 24 h and 72 h 1,25(OH)_2_D_3_ treatment are transcription factors; (2) over 22% of DEG after 24 h and 72 h of 1,25(OH)_2_D_3_ treatment are lncRNAs; (3) as much as 81% of the lncRNAs were upregulated after 24 h treatment with 1,25(OH)_2_D_3_, (4) all lncRNAs were dependent on the presence of the VDR protein in the cell, and more than 40% were dependent on the presence of the RXRA protein in the cell; (5) enrichment analysis on all DELs after 24 h and also 72 h treatment demonstrated that the most enriched motif was VDR: RXRA; (6) analysis of the protein partners for DELs in lncSEA platform demonstrated that their partners are mainly VDR.

Due to the limited data available on many long non-coding RNAs across different cancer types, predicting their precise functions following 1,25(OH)_2_D_3_ treatment in both healthy and cancerous skin cells remains a challenge. As summarized in Table 1 and Table 2, the lncRNAs deregulated by 1,25(OH)_2_D_3_ treatment of A431 SCC cells do not demonstrate predominantly anticancer properties across various cancers. It is important to highlight, however, that most of these lncRNAs have not been previously studied in the context of squamous cell carcinoma (SCC), and data regarding lncRNA expression in SCC are generally scarce. Interestingly, when the results of this study were compared with available lncRNA data from head and neck squamous cell carcinoma (HNSC), over 90% of the lncRNAs deregulated by 1,25(OH)_2_D_3_ in A431 cells were not typical for HNSC. This observation provides a valuable starting point for further investigations into the role of lncRNAs in modulating the genomic response of SCC cells to 1,25(OH)_2_D_3_ and their potential involvement in shifting the balance between pro- and anticancer lncRNAs.

To further explore the functional significance of lncRNAs in this context, we focused on a subset of candidates with potential links to interferon gamma signaling, a pathway known to be suppressed by 1,25(OH)_2_D_3_. Among the lncRNAs identified in our study, NPSR1-AS1, AATBC, and LCAL1 were selected for further qPCR validation based on in silico predictions indicating their potential involvement in the regulation of IFN-γ-related pathways. This selection was guided by gene ontology enrichment analyses, which showed that IFN-γ signaling pathways were significantly represented among both differentially expressed genes (DEGs) and lncRNAs (DELs) following 1,25(OH)_2_D_3_ treatment in A431 cells. Given that 1,25(OH)_2_D_3_ is well known to suppress IFN-γ signaling, these lncRNAs are of particular interest as potential mediators of this effect.

NPSR1-AS1 has been identified as a significant player in various cancers, underscoring its broad oncogenic potential. In lung adenocarcinoma (LUAD), NPSR1-AS1 is part of an eight-lncRNA signature associated with prognosis and immune infiltration, reflecting its potential as both a prognostic biomarker and a modulator of tumor immunity [69,101]. In thyroid cancer, NPSR1-AS1 promotes malignant behavior by stabilizing NPSR1 mRNA through interaction with the RNA-binding protein ELAVL1 (ELAV Like RNA Binding Protein 1), leading to MAPK (Mitogen-activated protein kinase) pathway activation [68]. Similarly, in hepatocellular carcinoma (HCC), NPSR1-AS1 is induced by hypoxia and drives proliferation and glycolysis via the MAPK/ERK pathway [67]. Its overexpression has also been linked to poor prognosis in virus-associated HCC, where it is part of a prognostic lncRNA signature correlated with survival outcomes [67]. In colorectal cancer, NPSR1-AS1 was validated as a diagnostic biomarker capable of distinguishing tumor tissue from normal samples with high accuracy [102]. Collectively, these findings highlight NPSR1-AS1′s multifaceted role in tumor progression, involving the regulation of proliferation, metabolism, and immune responses across cancer types. These mechanisms, particularly the activation of MAPK signaling and modulation of immune pathways, support the hypothesis that NPSR1-AS1 may also contribute to interferon gamma pathway regulation, a key target of 1,25(OH)_2_D_3_-mediated immune modulation in squamous cell carcinoma.

AATBC was identified as the second lncRNA deregulated by 1,25(OH)_2_D_3_ in A431 cells. AATBC (Apoptosis-Associated Transcript in Bladder Cancer) is a multifunctional lncRNA involved in metabolism and cancer. It enhances mitochondrial function and thermogenesis in adipocytes [103]. AATBC promotes metastasis in nasopharyngeal carcinoma via the miR-1237-3p–PNN–ZEB1 axis [104], and cervical cancer progression through miR-1245b-5p sponging [76] (It facilitates breast cancer migration via YBX1 and the YAP1/Hippo pathway [77], and drives prostate cancer cell proliferation through the miR-1245b-5p/CASK axis [75]. In bladder cancer, AATBC promotes proliferation and inhibits apoptosis by activating JNK signaling [78]. It is also upregulated in lung cancer, indicating diagnostic potential [105], and forms part of a prognostic lncRNA signature in melanoma [106]. In breast cancer patients, AATBC was analyzed alongside VDR- and ESR1-associated lncRNAs, showing correlations with histological grade [107]. Moreover, AATBC was included in a melanoma prognostic model related to gene instability, where its knockdown reduced melanoma cell proliferation and invasion [108]. Our findings suggest that AATBC expression is 1,25(OH)_2_D_3_-responsive in A431 cells; however, its functional role in SCC remains unknown. Notably, the lnc2cancer database does not report AATBC overexpression in HNSCC, and no published studies to date have explored its role in this cancer type.

LCAL1 was selected as the final deregulated lncRNA following 1,25(OH)_2_D_3_ treatment in A431 cells, and in silico analysis suggested that it may influence genes related to IFN-γ signaling. LCAL1 has been identified as a key regulator in lung cancer, where it supports tumor growth by inhibiting AMPK-related antitumor pathways [109]. It was originally described as one of 111 differentially expressed lncRNAs in lung adenocarcinoma and squamous cell carcinoma, implicated in cell proliferation and oncogenic signaling [110]. Moreover, LCAL1 forms part of an autophagy-related lncRNA prognostic signature in esophageal squamous cell carcinoma, underlining its broader relevance across cancers [111]. Only three studies mentioning LCAL1 were found in PubMed, highlighting the current limited knowledge on this lncRNA. Similar to the other lncRNAs studied, no data are currently available regarding the role of LCAL1 in SCC. However, database analyses, such as lnc2cancer, indicate that LCAL1 is not overexpressed in HNSCC.

In conclusion, this study demonstrates a strong time-dependent effect of 1,25(OH)_2_D_3_ in deregulating the expression of non-coding RNAs in the skin cancer cell line A431, while in normal keratinocytes (HPEKp), the response was markedly different, with a greater emphasis on transcription factors rather than lncRNAs. It can be speculated that at least some of the deregulated lncRNAs are part of secondary or indirect response pathways to 1,25(OH)_2_D_3_, potentially involving PDIA3-dependent and RXRA-independent mechanisms. Notably, LCAL1 appears to be RXRA-dependent, while AATBC and NSPR may act via RXRA-independent pathways. Understanding both classical and non-classical 1,25(OH)_2_D_3_ signaling in cancer cells provides new insights into the potential anticancer properties of 1,25(OH)_2_D_3_ in skin cancer.

This study shows that the active form of vitamin D_3_ can strongly influence gene activity in human skin cancer cells over time. In cancer cells, vitamin D_3_ caused a significant increase in the production of long non-coding RNAs, while in healthy skin cells, the main effect was seen in genes regulated by traditional protein-based mechanisms.

These long non-coding RNAs appear to play a major role in how cancer cells respond to vitamin D_3_. Some of them may act through less well-known pathways, independent of the usual vitamin D receptor partners. Importantly, most of the long non-coding RNAs identified in this study are not commonly seen in head and neck cancers, which suggests a new and specific pattern of response in skin cancer.

Some of these molecules may also influence how cancer cells respond to signals from the immune system, especially those related to interferon gamma. This connection could help explain some of the known anticancer effects of vitamin D_3_.

In summary, this study reveals that long non-coding RNAs may be key players in the way vitamin D_3_ works in skin cancer, and they could be valuable targets for future research or treatment strategies.

## 4. Materials and Methods

### 4.1. Cell Lines, 1,25(OH)2D3 and IFNγ Treatment

The human squamous carcinoma cell line A431 was obtained from Synthego (Menlo Park, CA, USA) and cultured according to the protocol described in our previously published work [33]. A431 cells with *VDR* (A431 Δ*VDR*) or *RXRA* (A431 Δ*RXRA*) gene knockout were purchased as a cell pool from Synthego (Menlo Park, CA, USA). A cell pool was selected for clonal isolation as described previously [33]. After appropriate isolation, CRISPR/Cas9 knockouts were verified by Sanger sequencing (Oligo. pl, Institute of Biochemistry and Biophysics) and Western blot analysis (see [33]). Pooled juvenile donors of Human Primary Epidermal Keratinocytes (HPEKp) were purchased from CELLnTEC (Bern, Switzerland). The cells were cultured in Epidermal Keratinocyte Medium (CnT-07, CELLnTEC, Bern, Switzerland) containing a supplement mix (A, B, C), Bisphenol A (BPE), and gentamycin with low calcium (0.07mM). The TrypLE™ Express solution (Gibco, Life Technologies, Waltham, MA, USA) was used for trypsinization at about 80–90% confluency. 1,25(OH)_2_D_3_ was purchased from Sigma-Aldrich (St. Louis, MI, USA), and IFNγ from Bio-techne (Minneapolis, MN, USA). Stock solutions of 1,25(OH)_2_D_3_ were dissolved in ethanol and stored at −20 °C. 100 nM concentration of 1,25(OH)_2_D_3_ and 10ng/mL IFNγ were used in the experiments.

### 4.2. RNA Sequencing (RNAseq)

The procedure for RNA isolation, preparation for RNA sequencing, and bioinformatic processing of the genome sequencing results of A431 cells and their clones was described in the work published by our team [33]. The raw results were used for further analyses of lncRNAs and TFs. As with the A431 cells, RNA was isolated from HPEKp cells and subsequently prepared for sequencing.

### 4.3. Bioinformatics Analyses

The bioinformatic procedures used for the analysis of sequencing results were detailed in our team’s previously published work [33] and were applied analogously to the analysis of HPEKp cell sequencing data.

CRISPR-mediated knockouts were generated using synthetic guide RNAs (gRNAs) targeting all known splice variants of VDR and RXRA, designed via Synthego’s CRISPR Design Tool. Knockout A431 keratinocyte pools (>50% editing efficiency) were obtained from Synthego (Menlo Park, CA, USA). For clonal selection, cells were seeded sparsely in six-well plates, and individual colonies were isolated using cloning discs (Bel-Art, Wayne, NJ, USA). Colonies were transferred to 12-well plates, cultured, and screened for gene disruption by Sanger sequencing (Oligo.pl), qPCR, and Western blots (Western blot for A431 ΔVDR—Figure S6, for A431 ΔRXRA in [33]; for A431 ΔPDIA3 in [21].

Raw sequencing reads were assessed for quality using FastQC [112] and cleaned with Trimmomatic [113]. PCA analysis was used for assessing data variance and condition clustering. STAR [114] was used for read alignment to the GRCh38 reference genome, and read counts were generated using featureCounts 2.0. [115]. Differential gene expression was calculated in DESeq2 [116] with criteria of |log2FC| ≥ 1 and FDR < 0.05.

Gene ontology (GO) enrichment analysis was conducted using the R package 4.2.2. topGO [117], applying Fisher’s exact test to identify the biological functions associated with differentially expressed genes. Gene names were annotated with GO terms using the org.Hs.eg.db package. Only GO terms with a *p*-value < 0.05 were considered significantly enriched. The results of the analysis were visualized using the R package [118].

Following the completion of bioinformatic processing, subsequent analyses were conducted using various online tools. Venn diagrams were made in the InteractiVenn program (http://www.interactivenn.net/, accessed on 1 July 2024); volcano plots were prepared with VolcaNoseR (https://huygens.science.uva.nl/VolcaNoseR/, accessed on 1 January 2024); visualizations of the DEGs on chromosomes were made in Phenograms (http://visualization.ritchielab.org/phenograms/plot, accessed on 1 January 2024); data preparation was conducted using BioMart Ensembl (https://www.ensembl.org/info/data/biomart/index.html, accessed on 1 January 2024). Gene descriptions and functional annotations were obtained using the DAVID tool (https://davidbioinformatics.nih.gov/, accessed on 1 January 2024). RNA–protein interaction enrichment analyses and gene set enrichment analyses were performed using the lncSEA 2.0 platform (https://bio.liclab.net/LncSEA/ accessed on 1 January 2025). Position frequency matrices for transcription factors were downloaded from the JASPAR database (https://jaspar.genereg.net/ accessed on 30 January 2024). To analyze differentially expressed lncRNAs after 1,25(OH)_2_D_3_ treatment, data from the HNSC section of the lnc2Cancer 3.0 database (http://bio-bigdata.hrbmu.edu.cn/lnc2cancer/, accessed on 30 January 2024)) were used. Genomic targets of lncRNAs were identified using the lncRNAfunc tool (https://ccsm.uth.edu/lncRNAfunc/, accessed on 1 January 2025). RNA-seq data generated as part of this work are available without restriction. The data have been deposited in SRA under accession number PRJNA926032 (transcriptomic results from HPEKp added to transcriptomic results from A431).

### 4.4. Analysis of the Transcription Factor Binding Sites and Their Enrichment Analysis

Transcription factor binding sites (TFBS) were predicted using CiiiDER (http://ciiider.com/ https://ciiider.erc.monash.edu/downloads.html accessed on 25 May 2024) [119]. Transcription factor binding sites (TFBS) were predicted also using CiiiDER (Hudson Institute of Medical Research, Clayton, VIC, Australia) (32). Gene sequences were scanned with VDR/RXRA position frequency matrices or the complete JASPAR matrix set. Promoter regions were defined as ±500 kb relative to the transcription start site and analyzed against the GRCh38 human genome assembly. TFBS prediction employed a default deficit value of 0.15, accepting matches with a score ≥ 0.85. Enrichment analysis was performed to identify transcription factors significantly over- or under-represented in differentially expressed genes (DEGs) relative to a background set. A detailed description of the methodology is provided in this work [120].

### 4.5. Real-Time qPCRs

All analyzed cell lines were treated with 100 nM 1,25(OH)_2_D_3_ for 24 h, or with a combination of 100 nM 1,25(OH)_2_D_3_ and 10 ng/mL IFNγ for 24 h, depending on the experimental conditions. Total RNA was extracted using the ExtractMe Total RNA Kit (Blirt, Poland) with on-column DNase treatment, according to the manufacturer’s instructions. RNA concentrations were determined using an Epoch Microplate Spectrophotometer (BioTek, Winooski, VT, USA). The reverse transcription was performed using the RevertAid First Strand cDNA Synthesis Kit (Thermo Fisher Scientific, Waltham, MA, USA). Quantitative real-time PCR was conducted on a StepOnePlus™ Real-Time PCR System (Applied Biosystems, Life Technologies, Grand Island, NY, USA) using the AMPLIFYME SG No-ROX Mix (Blirt, Gdansk, Poland). All primers were purchased from Merck (Darmstadt, Germany), and their sequences and groupings are provided in Supplementary Table S3. Gene expression levels were normalized to the reference gene RPL37 and calculated using the comparative ΔΔCT method. Results are presented as fold change ± standard deviation [39].

### 4.6. Immunofluorescence Analysis

Cells were seeded in eight-well imaging chambers (MoBiTec, Hamburg, Germany) at 200,000 cells/well, incubated overnight (37 °C, 5% CO_2_), and treated with 1,25(OH)_2_D_3_ in DMEM without phenol red, supplemented with 2% charcoal-stripped FBS, 2 mM L-glutamine, and 100 U/mL penicillin/streptomycin. After treatment, cells were washed with PBS, fixed with 4% formaldehyde (10 min, RT), permeabilized with 0.1% Triton X-100 (5 min, RT), and blocked with 10% BSA (1 h, RT). Primary antibodies (anti-STAT1, sc-136229; anti-p-STAT1 (Y701), sc-464; Santa Cruz Biotechnology, USA; anti-VDR sc-13133, Santa Cruz Biotechnology, USA; anti-RXRA A19105, ABclonal, Woburn, MA, USA) were applied overnight at 4 °C, followed by secondary antibody staining (Alexa Fluor 549 anti-mouse A21203, Invitrogen, Carlsbad, CA, USA; Alexa Fluor 488 anti-rabbit A11008, Invitrogen, USA; 1 h, RT), DAPI counterstaining, and mounting with DAKO fluorescence medium (S3025, Agilent, Santa Clara, CA, USA). The immunofluorescence stainings were performed in three independent replicates, analyzing 10–15 cells per condition. Imaging was performed using an Olympus Cell-Vivo IX83 microscope equipped with an ORCA-FLASH 4.0 camera and a 60× objective. Fluorescence intensity was analyzed using CellSens software version 4.3 (Olympus, Tokyo, Japan) with neuronal network-based recognition after 25,000 training iterations. Graphs were generated using GraphPad Prism 6.03 (GraphPad Software, San Diego, CA, USA).

## Data Availability

The data presented in this study are available on request from the corresponding author.

## Supplementary Materials

The following supporting information can be downloaded at: https://www.mdpi.com/article/10.3390/ijms26146632/s1.

## Abbreviations

BARX1	BARX Homeobox 1
BCL6	B-Cell CLL/Lymphoma 6
BNIP3P1	BCL2/Adenovirus E1B 19kDa Interacting Protein 3 Pseudogene 1
CALML3	Calmodulin Like 3
COLCA1	Colorectal Cancer Associated 1
CYP2B7P	Cytochrome P450 Family 2 Subfamily B Member 7 Pseudogene
DE	Differentially Expressed
DUXAP10	Double Homeobox A Pseudogene 10
EGFR	Epidermal Growth Factor Receptor
ELF4	E74-Like ETS Transcription Factor 4
EP300	E1A Binding Protein P300
FAM30A	Family With Sequence Similarity 30 Member A
FBS	Fetal Bovine Serum
FC	Fold Change
FDR	False Discovery Rate
FTH1P3	Ferritin Heavy Chain 1 Pseudogene 3
GAS5	Growth Arrest Specific 5
HDAC1	Histone Deacetylase 1
HMGN2P46	High Mobility Group Nucleosomal Binding Domain 2 Pseudogene 46
HNSCC	Head and Neck Squamous Cell Carcinoma
HOTAIR	HOX Transcript Antisense RNA
JUN	Jun Proto-Oncogene
LHX6	LIM Homeobox 6
lncRNAs	Long Non-Coding RNAs
MALAT1	Metastasis-Associated Lung Adenocarcinoma Transcript 1
MAPK/ERK	Mitogen-Activated Protein Kinase/Extracellular Signal-Regulated Kinase
NFE2	Nuclear Factor Erythroid 2
NR2F2	Proteins Nuclear Receptor Subfamily 2 Group F Member 2
OSR2	Odd-Skipped Related Transcription Factor 2
PDIA3	Protein Disulfide Isomerase A3
PDIA3P	Protein Disulfide Isomerase Family A Member 3 Pseudogene 1
PHBP1	PHB1 Pseudogene 1
PI3K-AKT	Phosphoinositide 3-Kinase/Protein Kinase B
PICSAR	P38 Inhibited Cutaneous Squamous Cell Carcinoma-Associated lncRNA
POU2AF1	POU Class 2 Homeobox Associating Factor 1
POU4F2	POU Class 4 Homeobox 2
PRAF2	PRA1 Domain Family Member 2 Protein
PTENP1	Phosphatase And Tensin Homolog Pseudogene 1
PTTG3P	Pseudogenes Pituitary Tumor-Transforming 3
RNA28S5	28S Ribosomal 5
RXRA	Retinoid X Receptor Alpha
SCC	Squamous Cell Carcinoma
SLC25A47P1	Solute Carrier Family 25 Member 47 Pseudogene 1
TFs	Transcription Factors
TINCR	TINCR Ubiquitin Domain Containing
TUSC2P	Tumor Suppressor Candidate 2 Pseudogene 1
VDR	Vitamin D Receptor
VDREs	VDR Binding Sites
WT	Wild-Type Cells
ZNF136	Zinc Finger Protein 136
