# Supplementary figures and images for: Vitamin D Reshapes Genomic Hierarchies in Skin Cells: lncRNA-Driven Responses in Carcinoma Versus Transcription Factor-Based Regulation in Healthy Skin
Source: Int J Mol Sci. 2025 Jul 10;26(14):6632. doi: 10.3390/ijms26146632 (PMC12296165; doi:10.3390/ijms26146632)

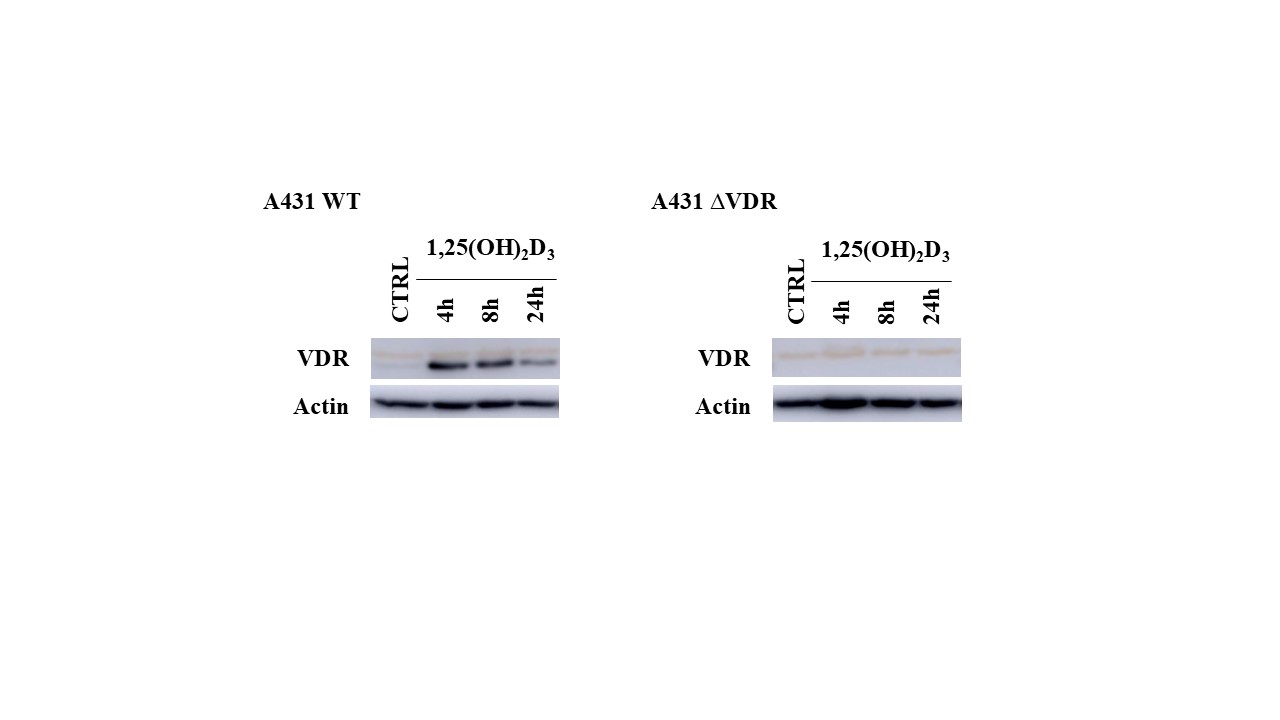

Supplement: Supplementary file 1 [file ijms-26-06632-s001.zip › Supplemental data S6.jpg]
